# Relevant Cybersecurity Aspects of IoT Microservices Architectures Deployed over Next-Generation Mobile Networks

**DOI:** 10.3390/s23010189

**Published:** 2022-12-24

**Authors:** Constantin Lucian Aldea, Razvan Bocu, Anca Vasilescu

**Affiliations:** Department of Mathematics and Computer Science, Transilvania University of Brasov, 500036 Brașov, Romania

**Keywords:** cybersecurity, IoT, microservices architecture, 5G network, energy monitoring

## Abstract

The design and implementation of secure IoT platforms and software solutions represent both a required functional feature and a performance acceptance factor nowadays. This paper describes relevant cybersecurity problems considered during the proposed microservices architecture development. Service composition mechanisms and their security are affected by the underlying hardware components and networks. The overall speedup of the platforms, which are implemented using the new 5G networks, and the capabilities of new performant IoT devices may be wasted by an inadequate combination of authentication services and security mechanisms, by the architectural misplacing of the encryption services, or by the inappropriate subsystems scaling. Considering the emerging microservices platforms, the Spring Boot alternative is used to implement data generation services, IoT sensor reading services, IoT actuators control services, and authentication services, and ultimately assemble them into a secure microservices architecture. Furthermore, considering the designed architecture, relevant security aspects related to the medical and energy domains are analyzed and discussed. Based on the proposed architectural concept, it is shown that well-designed and orchestrated architectures that consider the proper security aspects and their functional influence can lead to stable and secure implementations of the end user’s software platforms.

## 1. Introduction

The concept of Microservices Architectures (MSA) essentially relates to the larger agile developer professional community. It is interesting to note that both industry and academia have contributed to the development of the related research field [1], in an attempt to extend the capabilities of the classical Service-Oriented Architecture (SOA). Nevertheless, the effective relationship between these two architectural paradigms remains a matter of debate [2]. Thus, some proponents of MSA assert that it represents a new architectural philosophy, while some advocates of SOA almost postulate that MSA merely represents an implementational variation of SOA. The details of this intellectual debate are complemented by a sufficient understanding of Newman’s architectural principles [3].

In principle, MSA proposes the functional and logical decomposition of the application into several services, which are featured by a smaller footprint. They communicate through efficient mechanisms such as Representational State Transfer (RESTful) application programming interfaces (API) or stream-based data transmission models [1]. Usual principles and concepts [2] in the realm of MSA are “componentization via services”, which enhances modifiability, scalability, and deployability, “organized around business capabilities”, which ensures that the code is easy to read, follow and maintain “infrastructure automation”, which determines the continuous delivery processes and supports the activities of Software Development IT Operations (DevOps), and also “Decentralized governance and data management”, which favours flexibility and suitability. It is worth mentioning that relevant business conglomerates, such as Netflix, Amazon, and eBay, have chosen MSA in order to implement their sensible IT infrastructures.

Through this paper, theoretical and applied research results are used in the proposed architecture and its implementation by considering safety aspects for the development and deployment of a microservices ecosystem and these microservices’ internal structure.

One of the base ideas behind microservices is having smaller scoped units of work. A large system covering a big spectrum of business domain problems must be decomposed into smaller units of work. One distinguishes between different types of services: (a) data services that provide data, (b) business services, which offer more complex operations based on multiple data sources and (c) data types, translation services (facade services), and edge services that are used to deliver content to third-party contracts. Additionally, considering the microservice architectural styles, the monolithic artefacts are avoided.

Among other important advantages of a microservices-based architectural design, we can mention bounded contexts, fault tolerance, and governance flexibility [4]. Thus, bounded contexts imply that microservices are independent of one another, which means that they are interconnected only using the exposed endpoints of other microservices. Fault tolerance results from their capability to be dynamically loaded and multiplied based on the process load and can be achieved through the usage of circuit breakers patterns. These allow for the flow to continue even if errors or availability problems occur.

Microservices are deployed and afterwards operated on platforms that include hardware, software, and various types of services. The operation phase of the microservices brings multiple advantages such as independence, containerization, fault isolation, scalability, reliability, and updateability. While the monolithic application can be scaled only through a full redeployment of the whole application, the microservices can be scaled individually based on operational needs. Considering the cloud-native development style of the microservices, the application operates in cloud or outside of it while being portable and scalable. The application services can scale based on the load. Nonetheless, using this pattern, the application can also be run in a single data centre. Even if the microservices architecture and cloud-native development pattern are closely related to each other, it is possible to use them separately. That means that it is possible to create cloud-native applications without using microservices or microservice-based applications that are not built for cloud-native platforms.

**Figure 1:**
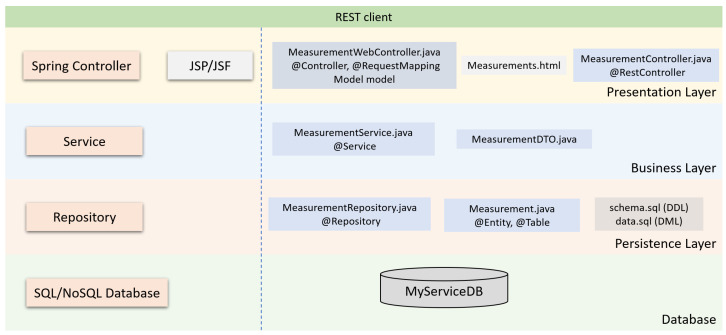
Multilayered architecture for shared data service.

Both web applications and other application types, such as legacy or mobile, are directly using the multi-tier architecture. The overview of the application architecture is split into tiers (Figure 1), making at least the distinction between these tiers, as follows:

- The presentation layer covers a broad space of graphical user interface technologies and frameworks. Different types of end devices (computers, phones, PDAs) support different types of UIs. Some of the stakeholders are using the SAP UIs, while other parts are using HTML5 UIs based on React, Angular, or another JavaScript platform. Due to the continuous evolution of the UI packages, the adoption of the microservices architectural style helps when the UI becomes obsolete by allowing the replacement of single defective components without having to redefine all the UIs for all the clients;
- The business layer (BL) is compounded from the majority of the composite microservices. The BL services usually require scaling up;
- The persistence layer implements the storage and retrieval of the data. It translates telegrams from the event queues and persists their content. It allows uniform queries when information is requested. The data sources tier works with all kinds of data sources: relational databases, semi-structured JSON data, smart meter gateways APIs, and NoSQL databases, which are used for the storage of large amounts of measurements, and their historical evolution.

The microservices architecture exploits the tier architecture and adds multiple services for each of the initial tiers having the possibility to be scalable through the addition of new integration patterns like gateway patterns, edge patterns, and process aggregator patterns. Due to the data complexity, multiple data sources need to be managed and simultaneously used by the application and foreseen during the software system’s design phase. One can use single-service databases, shared services, asynchronous events, or other data access-related technologies. In the case when services represent multiple layers, a precedence relations hierarchy can be organized into tiers. However, if the dependencies have a deep mesh structure, then the microservices’ interdependence graph organization can be built, and if needed, subsets of the interrelation graphs can be treated as architectural tiers.

Despite that, the services can be grouped on levels based on their common logic, and they need to consider and enforce cybersecurity rules such as the zero trust principle or security in-depth principle. It is not good practice to build trusted networks containing microservices groups, even if they need to exchange information intensively. Since the services are distributed in nature, the coupling must be carefully controlled.

Due to their scalability and granularity, microservices are an enabler for the rapid development and deployment of secure applications while also leveraging the limitless resources of cloud providers.

Identity providers are a good solution for creating and managing access tokens. They provide permission management, profile management, roles, permission delegation, registration, and credential management. Additionally, they are compatible with existing security standards like OAuth [5]. Some open-source solutions can be customized and integrated into the application and used as gateway microservices in order to implement the authentication mechanisms and increase overall security.

It is generally commonplace to assert that the Internet of Things (IoT) allows humans and computer systems to interact with billions of devices or data collection IoT appliances, such as sensors, actuators, services, and other gadgets that are featured with an Internet connection, or another kind of data interface. It is immediately noted that IoT devices are placed between cyber and physical environments, which significantly improves the general human capacity to interact with the environment. From this point of view, the research IoT approach could face cybersecurity problems by harnessing the properties of its own devices and information and communication technologies (ICTs) [6] or could consistently benefit from artificial intelligence support for efficient solutions [7,8]. Middleware systems or software layers, which are commonly defined as a software system designed to intermediate the link between IoT devices and related applications, represent a fundamental technology. Therefore, the proposed IoT middleware architectures must comply with the requirements of the related IoT ecosystem to be adopted as a proper solution on a larger scale. The service-oriented architecture (SOA) represents one of the approaches that is particularly considered. In this context, the particular type of service-oriented paradigm, which is represented by microservices, has naturally built a hype in the realm of cloud and enterprise-oriented applications. Moreover, the microservices model is particularly convenient relative to dynamic IoT applications, which usually consider architectures that are based on the usage of microservices. The contribution that is reported in paper [9] represents an overview regarding the practice and use of IoT microservices architectures. Thus, the authors assess the requirements of a typical IoT middleware through an investigation that addresses the requirements for the design and implementation of the appropriate middleware architectures.

Additionally, the interested reader may consult the rather extensive review concerning the broader scope of microservices architectures security, which is presented by the authors of [10,11].

The contribution that is described in this paper considers microservices as basic pillars of the respective system’s architecture, which is concerned with the efficient and secure management of tens of thousands of electricity meters. The microservices use standard TCP data channels in order to exchange data between them and also with other system components. Nevertheless, for the TCP-based traffic, some use-case scenarios require to be translated to DNP3-based data traffic [12], which is compatible with the specific Supervisory Control and Data Acquisition (SCADA) architectural components of the system. In Section 2.2, some of the most relevant existing contributions are analytically enumerated.

The rest of the paper is structured according to the following sections. Significant existing papers are presented in Section 2.1, which also pertain to the scope of understanding the supervisory control and data acquisition systems (SCADA) described in Section 2.2. Furthermore, the shared data microservice is thoroughly described in Section 3, from an architectural and implementational perspective. Moreover, the authentication microservice (see Section 4) is also presented and evaluated. In the next Section 5, further security aspects for the energy platform related to the microservices that consider IoT devices are presented and analysed. Consequently, the real-world significance of the approach that this paper proposes is evaluated and discussed. The last section concludes the paper.

## 2. Relevant Existing Contributions

The consistent developments that have been realized in the scope of next-generation mobile networks mandate their consideration as a viable solution for the creation of complex energy-efficient infrastructures, which ensure that the data are optimally transmitted between the various IoT physical devices, and the proper microservices. Therefore, it is immediate to infer that there are interesting scientific contributions that are reported in the relevant literature.

Thus, the contribution that is reported in [13] is concerned with the evaluation of deployment models for services in 5G networks, where the network functions (NF) are designed and deployed through the consideration of traditional architectures, where the functions are specified in a virtual machine (VM), or through the consideration of serverless architectures, which suggests that the functional features are distributed in containers. Additionally, the effective performance of microservices in Kubernetes is also assessed. The evaluation that is performed also considers the employment of different versions of the HTTP protocol, which are used to implement the service-based interfaces of the 5G services. The authors’ results reported here demonstrate certain performance benefits through the utilization of HTTP/3, which sends data using the QUIC transport protocol [14] in scenarios with networks characterized by losses or delay conditions. Nevertheless, deployment in 5G networks must carefully consider aspects that are related to connection tracking mechanisms, which should scale up relative to a high volume of requests.

The authors of [15] propose a 5G satellite edge computing framework, which they abbreviate as 5GsatEC. It aims to reduce the delay and expand the network coverage. This framework consists of embedded hardware platforms and edge computing microservices that should be applied to the design and deployment of satellites. The increased flexibility of the framework relative to complex scenarios should be provided by the consolidation of the resources management at the level of the central processing unit (CPU), graphics processing unit (GPU), and field-programmable gate array (FPGA). They consider three types of services: system services, basic services, and user services. The assessment of the proposed system’s performance is conducted using a series of experiments. The reported results show that 5GsatEC provides a broader coverage than the 5G network. The results that are presented claim that 5GsatEC provides a lower delay, packet loss rate, and bandwidth consumption relative to the 5G satellite network, which should be expected, in principle. Nevertheless, this suggests that critical microservices-based architecture can be adequately sustained by such an infrastructure.

The scientific contribution that is reported in [16] introduces SWEETEN, which is a system that potentially assists the user in operating a 5G network using adequate management tools in a transparent manner to the user. The enrichment of the related functional stack through the addition of high-level management features implies that the users may readily deploy an augmented services stack that includes both network-related operational features and management functions. Thus, the authors present a prototype, which is evaluated using a dynamic Cloud Radio Access Network (C-RAN) [17]. The experimental results they report confirm that SWEETEN can assist users with the easy deployment of complex management solutions.

The flexible and open architecture of 5G networks needs to accommodate a variety of services. Consequently, the current open architecture microservices models, which manifest in the scope of 5G and next-generation mobile networks and are launched by the current industry, require establishing the technical standards for the proper integration with the industrial Internet. They also require solving the adaptability problem between the packet scheduling mechanism that relates to the User Plane Function (UPF) [18] at the edge of the data network, thus increasing the efficiency of the overall data infrastructure’s usage. Thus, the authors of paper [19] specify a 5G microservices open model through the consideration of the object-oriented modelling method in the scope of the industrial Internet. Thus, considering the IEC61850 standard [20], common services such as clock and location inside the 5G networks are defined as a public data class. These constitute the microservices that support various logical functions inside the 5G network as well as Virtual Network Functions (VNF) [21,22] calls outside the 5G network, which improves the reuse and consistency of the 5G-related microservices. Furthermore, the pseudo time synchronization flow scheduling algorithm, which is related to UPF, is designated to realize the matching between the time-deterministic service of industrial Internet [23], and the asynchronous packet scheduling carried by UPF switches and clustering techniques, which ensures the deterministic and smooth service bearer of industrial Internet [24,25]. The simulation experiment shows that the proposed algorithm can improve the time accuracy of the 5G business model and improve the time, punctuality, and coordination of user plane bearing under the microservice 5G capability open architecture.

The solution that is described in [26] identifies and leverages the coupling relationship between computing and network fabric for various microservices, and it solves an optimization problem to appropriately identify how each microservice should be deployed in the complex, multi-tiered compute and network fabric, so that the end-to-end application requirements are optimally satisfied. The authors report two real-world 5G applications relative to the video surveillance and intelligent transportation system (ITS) domains. They suggest that ROMA, the described solution, can save up to 90%, 55% and 44% of the computational resources and up to 80%, 95% and 75% of the network bandwidth for the surveillance (watch list) and transportation application (person and car detection), respectively. Nevertheless, this represents a solution that performs in a decent fashion considering particular use cases, and it is difficult to predict its appropriateness for other real-world use case scenarios.

Moreover, it is interesting to note that the paper [27] concentrates on a description of a 5G platform-oriented solution among different approaches, which is used to integrate authentication and authorization functionalities, a claimed secure and stateless mechanism, and also, the provision of identity and permissions management, which are used in order to handle not only users, but also system microservices in a network functions virtualization management and orchestration (NFV MANO) system [24] that is oriented towards the deployment of virtualized services. The solution that is described considers the NFV-based SONATA Service Platform [28], which offers functional specifications that are necessary for a continuous integration and delivery methodology, which offers high levels of programmability and flexibility that are useful in order to manage the entire life cycle of Virtual Network Functions. Moreover, the authors claim that their approach is compatible with several authentication and authorization mechanisms, which can be used to manage the access of users to the relevant microservices in a 5G platform.

Furthermore, the contributions that are reported in [29] consider an architecture that uses microservices to provide what the authors call a “finer scalability” and more effective resource usage than regular monolithic microservices architecture designs. Additionally, it is relevant to note that there are contributions that describe related models, such as the concept of artificial intelligence (AI) as a microservice (AIMS), which is considered a pretext by the authors of the paper in [30]. The respective architecture was designed to support the design and development of AI microservices, which can be deployed on federated and integrated 5G networked slices in order to provide autonomous units of intelligence as blockchain-based systems for edge-of-things [31] or applied in the market sectors to a supply chain [32,33], as opposed to the current monolithic IoT-Cloud services. The proposed 5G-based AI system is envisioned as a platform for the effective deployment of scalable, robust, and intelligent cross-border IoT applications that are intended to provide enhanced levels concerning the quality of experience in scenarios where real-time processing, ultra-low latency, and intelligence are key requirements.

The authors of [34] describe an interesting 5G microservices deployment model, which combines software-defined networks (SDN) [35] and network functions virtualization (NFV) [36] to efficiently create (orchestrate) microservices on fully functional logical 5G data infrastructures.

The low-latency 5G and beyond networked infrastructures determine a consistent research scope. Thus, in [37], the authors describe a dynamic runtime that enables low-latency applications to use 5G data networks effectively. They design a runtime, which continuously monitors the communication that is established between the microservices, and it estimates the data that they exchange. The respective runtime also handles temporary network partitions, and it also maintains data consistency. It is relevant to note that this kind of scientific contribution is important, as the transmission of data between microservices should be conducted through low-latency data transmission channels. In [38,39], the relevant principle is analysed and extended for data networks of several types.

The design of low-latency models becomes increasingly important considering that the next-generation mobile networks are evolving from centralized to distributed architectures and from human interaction to AI-powered architectures in order to achieve the self-adaption to system dynamics [40]. Especially for these approaches, the cloud-native paradigm with services decomposed into microservices is particularly relevant. Thus, the distribution of the relevant network functions is important. In [41], the authors describe a microservice placement strategy, starting from the internal service composition to the particular communication model which is established between microservices. They regard the placement as an optimization problem with the aim of minimizing the end-to-end service latency. They address the optimization problem with a combination of greedy and genetic algorithms. Furthermore, interesting data traffic optimization models for microservices are introduced in [42,43].

Network slicing enables communication service providers to partition physical infrastructure into logically independent networks. Network slices must be provisioned to meet the service-level objectives (SLO) of different commercial services, such as enhanced mobile broadband, ultrareliable low-latency communications, and massive machine-type communications. Network orchestrators components have the role of customizing the service placement and the proper scaling model to obtain the necessary SLO for each network slice [44]. Moreover, the authors of the paper [45] describe the challenges that are encountered by network orchestrators concerning the allocation of the necessary resources to different 5G network slices. They also suggest the use of artificial intelligence to infer the core placement and scaling decisions, which meet the requirements of network slices that are deployed on shared infrastructures.

### 2.1. Software Platforms

Containers and microservices are regarded as one of the most natural approaches for deploying IoT applications in various cloud-based environments, for example, in the agricultural sector [46]. Nevertheless, a significant security problem determined by this model relates to the software containers that are not securely patched and which consequently produce vulnerable microservices. Although some existing contributions aim to reduce the implied security risks using vulnerability detection tools, outdated databases prevent a proper detection process to occur on newly published vulnerabilities. The research work reported in [47] describes a system intended to enhance container-side security models, which targets unknown attack patterns using a "mimic defence network". More precisely, a resource pool that contains attack pattern images is built. Following this, the variability of the execution outputs is analysed to detect potential unknown vulnerabilities. The possible continuous attacks are essentially mitigated through the usage of a graph-based scheduling strategy. This strategy optimizes the randomness and heterogeneity of the attack pattern images that are considered to replace the existing images. Additionally, a system prototype is implemented and described. The authors claim that the results of the experiments show that it is necessary to send 54.9% more random requests to complete the attack successfully. The authors also claim that the reported approach further enhances the defence success percentage by approximately 8.16%.

The integrated collection of personal health data represents a relevant research topic, which is enhanced further by the development of next-generation mobile networks that can be used to transport the acquired medical data. The gathering of personal health data has recently become feasible using relevant wearable personal devices. Nevertheless, these devices do not possess sufficient computational power and do not offer proper local data storage capabilities. The scientific contribution that is reported in [48] presents an integrated personal health metrics data management system, which considers a virtualized symmetric 5G data transportation system. The personal health data are acquired using IoT wearable devices [49] through a client application component, which is typically deployed on the user’s mobile device, regardless it is a smartphone, smartwatch, or another kind of personal mobile device. The collected data are secured and transported to the cloud data processing components using a virtualized 5G infrastructure and homomorphically encrypted data packages. The system has been comprehensively assessed through the consideration of a real-world use case, which is presented. The system is assessed through a field trial, which considers residents of Brasov City, Romania. The paper demonstrates that it is possible to design, implement and deploy a complex distributed system, which uses IoT devices for data collection, and implements complete end-to-end data privacy mechanisms through the consideration of homomorphic encryption [50] routines.

### 2.2. SCADA Architectures

In this section, some of the most relevant existing contributions regarding SCADA Architecture are analytically enumerated.

The contribution that is presented in [12] relates to an Intrusion Detection and Prevention System (IDPS), which is based on the Distributed Network Protocol 3 (DNP3). The proposed IDPS is called DIDEROT (Dnp3 Intrusion DetEction pReventiOn sysTem), and it uses both supervised Machine Learning (ML) and unsupervised/outlier ML detection models, which are able to infer whether a DNP3 network data flow is characteristic of a particular DNP3 cyberattack or anomaly. Thus, the supervised ML detection model is used, which attempts to assess whether a DNP3 network data flow is connected to a certain DNP3 cyberattack pattern. If the corresponding data pattern is deemed normal, then the unsupervised/outlier ML anomaly detection component is enabled, which aims to detect the presence of a possible problem. Considering the detection results of the DIDEROT system, the general Software Defined Networking (SDN) approach is chosen in order to efficiently mitigate the corresponding DNP3 cyberattacks and abnormal data traffic patterns. Nevertheless, the performance evaluation is not thoroughly conducted, which represents the main drawback of this paper.

The authors of [51] describe an intrusion detection system (IDS) for DNP3 networks, which they claim is effective in order to observe and monitor critical data transfer operations in DNP3-based systems. The presented anomaly detection model considers possible attack patterns, which may bypass any rule-based deep packet inspection once the attackers obtain access to the main server systems. The first step shows that the data sets are generated, which mirror the DNP3 data traffic features, which are observed in real-world power grid substations for a sufficiently long time. Furthermore, the input features are extracted, which are determined by the function codes per TCP connection, along with other relevant TCP characteristics. Furthermore, an unsupervised deep learning model, called Autoencoder, is used to learn the normal behaviour of DNP3 traffic based on function code patterns. The authors refer to their approach as FC-AE-IDS (Function Code Autoencoder IDS). The proposed model is evaluated considering three datasets. The experimental results suggest that the proposed model’s detection accuracy is approximately 95% in the case of all the attack scenarios that have been considered.

The authors of [52] presented two relevant ideas. Thus, the first idea suggests the improvement of the SCADA architecture security using asymmetric cryptography models as well as digital signatures. The overhead that is generated is assessed from a quantitative perspective. This mediates reaching certain goals, such as the specification of data-origin authentication and also the traceability and implicit non-repudiation of commands given to the smart field and direct control equipment. Furthermore, the possibility of implementing digital signatures with a minimum impact on a standard and reliable industrial data communication protocol, such as the Distributed Network Protocol version 3 (DNP3), has been evaluated, and the obtained results are presented. Additionally, the second main idea of this paper regards the design and development of a multitenant cloud-based architecture, which is compatible with a SCADA environment. This architectural hypothesis is centred towards SCADA operators that manage multiple industrial control systems (ICS). This approach also has the goal of consolidating process data in a centralized fashion.

The main contribution of [53] is to analyse the use of machine learning techniques comparatively in order to classify messages that conform to the same protocol, which are exchanged through encrypted tunnels. The study describes four simulated cases of encrypted DNP3 data traffic scenarios and also four different supervised machine learning algorithms. These are decision tree, nearest-neighbour, support vector machine, and naive Bayes. The results of the experimental research process suggest that it is possible to extend a Peekaboo attack [54] over multiple substations. This experimental setup relates to a decision tree learning algorithm, which gathers the relevant information from a system that communicates using encrypted DNP3 data traffic channels.

The valuable contribution of intelligent systems in the scope of the industrial domain is obvious. Thus, industrial automation processes, supervision, remote control, and fault reduction represent only some of the various real-world process optimizations which the relevant and intelligent technologies provide. The Distributed Network Protocol 3 (DNP3), as has already been mentioned, represents a multi-tier application layer protocol, which proves to be particularly useful in critical industrial settings, such as complex electrical grid systems. The authors of the paper [55] study the internal vulnerabilities which are induced by the design of the DNP3 data transmission protocol. They implement the attack patterns that are determined empirically, and the research process is experimentally documented through eight DNP3 attack scenarios. Moreover, they describe the architecture of a multi-model Intrusion Detection System (IDS), which is based on Deep Neural Networks (DNN). The proposed model is trained using synthetic experimental data, which the authors generate. The presented approach is compared with several machine learning algorithms, which are considered for data classification. The authors claim that the proposed system is able to detect DNP3 attack patterns with an accuracy of 99%. This is one of the reviewed papers, which clearly presents the advantages and potential problems of DNP3-based SCADA architectures, and also describes the basic features of general SCADA architectures.

## 3. Shared Data Access Microservice

This section presents practical steps that can be considered in order to create a data microservice. The development and testing of microservices consider several software platforms, as follows.

- Java SDK—the Java programming language software development kit. There are multiple Java variants available that can be used. The Java versions are implementations of the JSR 390 as specified in the Java Community Process. For example, for this sample, the OpenJDK [56] service distribution was used.
- Maven—build tool that helps the development process to easily manage the project files and the dependencies, which a project component uses [57].
- Eclipse—this is used in order to simplify code writing through the usage of an integrated development environment (IDE), an approach that is recommended. Among the most widespread IDEs that are used in the Java ecosystem, we may enumerate Eclipse, IntelliJ, and NetBeans. Relative to small projects, the use of an IDE is not very helpful, but for complex projects, the IDE, in tandem with the build tool, simplifies the software development process [58].
- PostgreSQ—relational database management system, which is used in order to store the data manipulated by the service [59].

### 3.1. Microservices Demo Project Measurements

The demo service application concept is a simple web application that is based on Spring Boot, which implements the microservice architecture, uses the Java Persistence API (JPA) specification, and also considers the server-side UI framework Thymeleaf. The Thymeleaf template engine allows the design and manipulation of a graphical user interface controls and events. It should be noted that Spring Boot can be used in order to develop serverless/independent applications or web applications, which are managed and run with the help of application servers.

Thus, Figure 1 presents the internal architecture of a single microservice. The internal architecture of a microservice is organized on multiple tiers, each tier with its own responsibilities. The consideration of the separation of concern principle implies that the microservice can be easily designed, coded, and improved, if required.

Being a simple service, a graphical user interface is also created next to the service, even if, in the production environments, this is not mandatory since the views are independently created and managed, and they are often based on different technologies. The service publishes a single endpoint only be accessed using the exposed endpoint.

**Figure 2:**
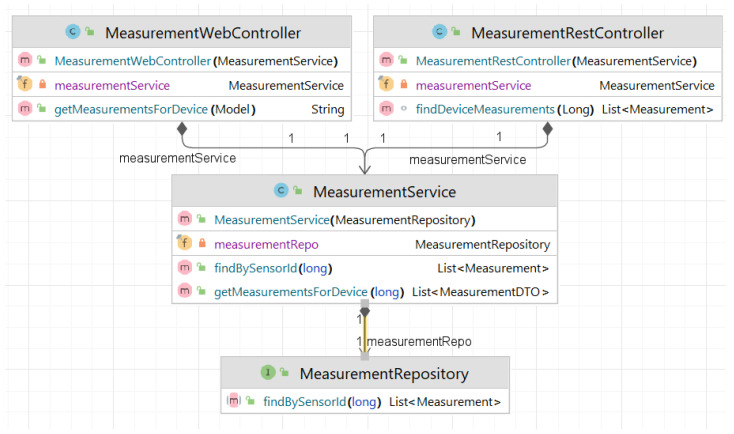
Class diagram for data service.

The creation of a data wrapper microservice, which is represented in Figure 2 that is compatible with the system that is described in this paper or with other similar software systems relates to the following steps.

S1 Generation of the Spring Boot project using the online tool;
S2 Import the skeleton project for further development into the Eclipse;
S3 Populate the database with data;
S4 Define an entity (Measurement entity class);
S5 Create a repository (services for working with entities);
S6 Create a business logic service;
S7 Implement the controller for using the service from step S6;
S8 Creation of a simple UI for direct service access using the Java-based *Thymeleaf* templates engine;
S9 Embedded server port configurations. Modify the application configurations by changing the connection port using the *application.properties* file;
S10 Define a REST controller for the service in order to work with it without a graphical interface.

### 3.2. S1 Generation of the Spring Boot Project

**Figure 3:**
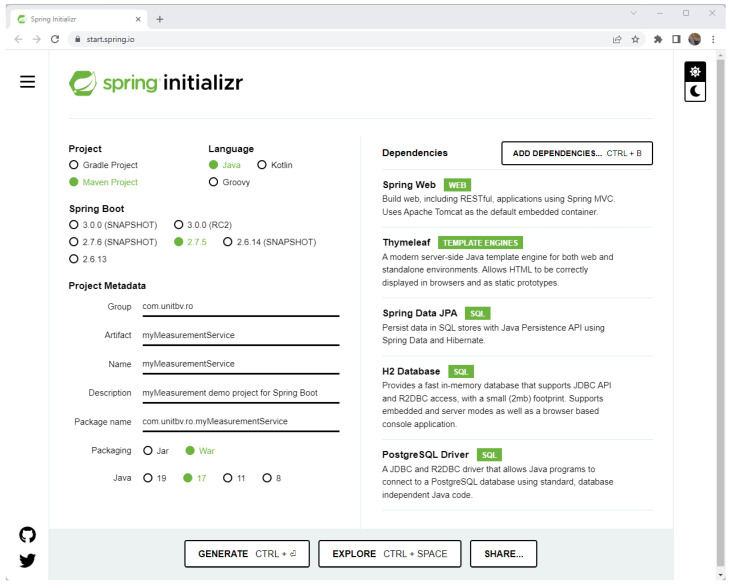
Use of the Spring Initializr for initial project setup.

Using the Spring Initializr [60], generating multiple types of projects is possible. It offers JVM-based skeleton projects, which are related to different tools for building software projects, like Maven or Gradle, and through the usage of a variety of programming languages such as Java, Kotlin, and Groovy. The utility tool Spring Initializr can be used through the command line or through the custom Web UI (Figure 3).

Some of the minimal settings that were selected for the current example are:

(1) ***Maven*** as a build tool.

Even if the developers use an IDE for writing code, the extended control of the project dependencies requires that they may constantly consult and correct the entries and their versions into the pom.xml file, which is created and maintained in the case of Maven projects.

Maven offers many archetype projects, and it is used inside integrated development environments such as Eclipse.

(2) Programming language selection: *Java*.

Although the ownership of Java software development kits has become more complicated nowadays, it is still a good programming language, which has support for many types of web technologies and web projects.

(3) Spring Boot version selection.

Over time, many technologies and libraries have been created in the Java ecosystem. It yields that, in order to build a complex web project, the user simply needs to combine them and maintain compatibility between their versions. Spring Boot was conceived as a framework that assists the developer in creating stand-alone projects easily.

(4) Project packing type selection: *war*.

A web application contains multiple classes, which can be easily handled by packing them into libraries and archives. An application archive may contain multiple other libraries. Spring Boot embeds the Tomcat web application server in order to run the project as a default Servlet container. By default, due to its configuration, it is possible for the application to be started as a Spring Boot application inside the Tomcat server. If the user wants to use another java Servlet container, the archive that contains the application must be deployed on that server. One of the first steps during the application deployment is represented by the unpacking of the application archive into proper installation directories. The created microservice is accessible through the defined endpoints, and it does not actually require the graphical user interface.

Considering that a simple web application has been created, and the WildFly web server (Servlet container) has been used for its deployment, the archive type war (web archive) is chosen.

(5) Select the targeted Java version for the project (in the current case, *Java 17*).

(6) Establish and select the dependencies (Web, Thymeleaf, JPA, database drivers, etc.)

The Java web projects use the existing Java libraries to create and add new functionalities to the business processes. Maven uses the dependency concept to describe a library or a library package with some functionalities already implemented, which the project depends on. For example, the Apache Log4j library contains the logging functions for handling log messages and log files, and it can be used as a Maven dependency. The generation of the necessary web project implies that the following dependencies are selected:

a. Spring Web—web application based on the Spring MVC framework;
b. Lombok—annotation library for avoiding boilerplate code;
c. Thymeleaf—server-side Java template engine;
d. Spring Data JPA—API for data persistence;
e. PostgreSQL Driver—Java SQL driver for connecting to PostgreSQL database server.

(7) Set the information needed to identify the project and project version using the project metadata tags:

- Group: *org.unitbv.ro*;
- Artifact: *myMeasurementService*;
- Name: *myMeasurementService*;
- Description: *myMeasurementService demo project for Spring Boot*;
- Package name: *org.unitbv.ro.myMeasurementService*.

Using the *GENERATE* button, the project generation is conducted, and the archive *MyMeasurementService.zip*, which includes the project structure, is created and automatically downloaded.

### 3.3. S2 Import of the Project into Integrated Development Environment Tool

**Figure 4:**
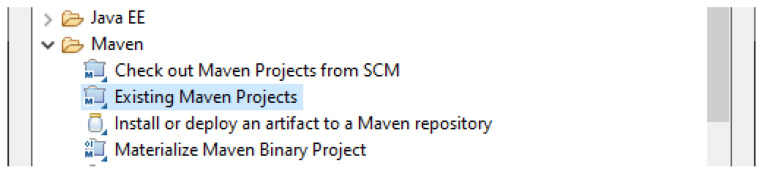
Importing existing MAVEN project.

The project generated with the Spring Initializr web tool is only a skeleton project for a web application that must be further developed. To simplify the code writing, the project can be imported into a proper IDE, such as Eclipse, by completing the following steps:

(1) From the file menu, select import and then import the existing maven project (Figure 4). Since the microservice is integrated into a broader distributed ecosystem that contains multiple services, it is recommended to use the maven archetypes to keep track of sources and folder structures in the same way.

(2) After the import is successfully completed, the Maven configuration file *pom.xml* is inspected in order to see the dependencies and the project’s meta data. These project configuration parameters can be extended or modified as needed.
(3) The initial main class of the web application can also be inspected and modified.
(4) Create an empty database on the database server (e.g., by using the SQL command *create database*).
(5) Specify the newly created database as the data source for the application into the *application.properties* configuration file (Listing 1).
(6) Check that the skeleton functionalities and project dependencies are properly set, and the application might be started (in Eclipse, Run As -> Spring Boot App).

**Listing 1.** Sample database connection settings using *application.properties* file.spring. jpa. hibernate. ddl − auto = nonespring. datasource. initialization − mode = alwaysspring. datasource. platform = postgresspring. datasource. url = jdbc:postgresql://localhost:5432/MyMeasurementServicespring. datasource. username = postgresspring. datasource. password = 1q2w3e

The Spring Boot App menu options are available in the IDE menu only if the Spring Tools for Eclipse were previously installed into the existing Eclipse instance using the Marketplace component.

If the application is available, and no syntax errors are detected, an embedded instance of the Tomcat web server is started on localhost, by default on port *8080*.

Another possibility is to generate the web archive as a *war file* by issuing the following command, inside or outside of the Eclipse IDE:

mvn clean install

If successful, the command will create the file *MyMeasurementService.war*, which can be further deployed or installed into the web container. Relative to the WildFly application server, the *war application* can be installed by simply copying it into the folder *standalone∖deployments*.

(7) For testing the application, it must be called by using its URL into a web browser http://localhost:8080/myMeasurementService.

**Figure 5:**
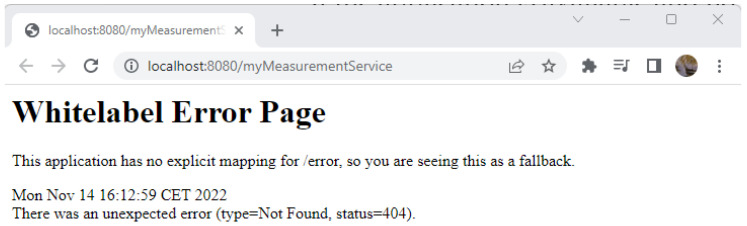
Whitelabel Error Page.

Since no modifications were made to the project, the *Whitelabel Error Page* message will be displayed (Figure 5). Consequently, a default error page is generated by the newly generated application.

The next steps can be followed in order to add a hello message landing page.

**Figure 6:**
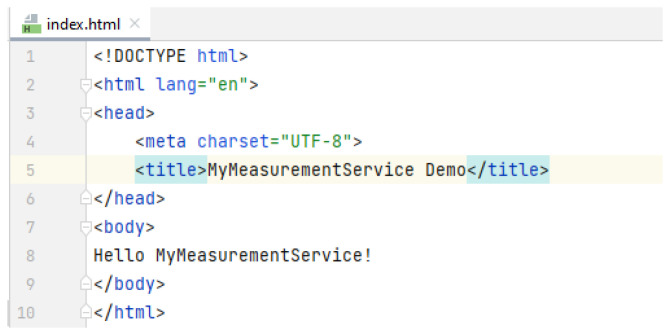
Static *index.html* file for the web application.

**Figure 7:**
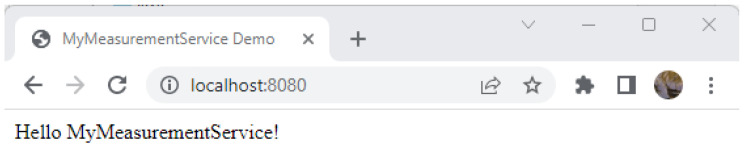
Welcome message page of the web application.

(a) Into the *resources∖static* sub-folder of the project, the *index.html* file can be created (Figure 6). The static pages have fixed content that is not modified by other scripts or components. In practice, the static pages are used only the forward the request to other dynamic pages.
(b) The content of the newly created file, *index.html* is customized by adding the hello message *Welcome to the MyMeasurementService test application* (Figure 6).
(c) After the recompilation and redeployment of the web application, the new page (index.html) content should be visible when the browser asks for the default page (Figure 7).

Note that the application can be manually started by issuing the following Maven command: *mvn spring-boot:run*.

### 3.4. S3 Sample MyMeasurementService Database Initialization

The database structure and the initial data can be developed in multiple ways. Among these:

1. Code-first approach—automatically generated by using the JPA annotations;
2. Database-first approach - create and populate the database by using some default SQL scripts. The default SQL script files, which are detected and interpreted by Spring Boot are schema.sql and data.sql. These two files are considered if they are present in the *src\main\resources* project folder.

In both cases, the database and the connection to it have to be specified in the *application.properties* project configuration file. The following steps need to be followed in order to create the database and initial data based on the scripts.

(a) Inside the *application.properties*, stop hibernate in order to generate the database schema by setting.
spring.jpa.hibernate.ddl − auto = none
  It should be noted that this command will stop Spring Boot from generating the database based on the annotations, and the schema.sql and data.sql files will be considered.
(b) Create an empty database on the database server (e.g., MyMeasurementService).
(c) Edit the database connection credentials for the project into the *application.properties* file, in order to connect it to the database server (e.g., PostgreSQL).
(d) Create the data definition language script file *schema.sql*. The SQL script with commands for the database structure initialization is located into the project folder *src/main/resources*. Listing A1 presents a sample *schema.sql* file.
(e) Populate the database with the initial data. A data file *data.sql* is created into the project folder *src/main/resources*. This file contains the SQL queries for inserting data into the database tables. Listing A2 contains sample data for initializing the database.

Considering the provided measurements, the id = 100 represents a blood pressure meter, and its corresponding unit is mm-Hg (unit id = 20) with two readings per day. The measures can be of different types (systolic and diastolic). The measurements of the device with id = 300 can represent a smart meter gateway reading having Watt (unit id = 75) as consumption unit and as type, the tariff type (e.g., based on readings frequency, for example, monthly readings).

(f) Restart or redeploy the application, and verify the possible error messages displayed on the server console.
  Notes:
  - It is possible to use spring. jpa. hibernate. ddl − auto = create − drop to force JPA to delete and recreate the database and initial data for each application redeployment/restart;
  - Use drop commands to remove the tables and table data if they are not properly generated when starting the project (drop views, tables, sequences, etc.), or remove the database and recreate it as an empty database with the command drop table measurements;
  - The drop order is important: firstly, one must delete the tables that use foreign key related data, then the tables with the corresponding primary key data are deleted;
  - When inserting the initial data, the order must also be considered in the sense that we cannot create a measurement without having its device already inserted; this rule must be considered when creating the *data.sql* file;
  - When restarting the application, the database schema must be (manually) deleted so that the JPA/Spring Boot component will be able to recreate it or the setting spring. jpa. hibernate. ddl − auto = update could be used.

(g) Each time when needed, the database status can be inspected using the default sql client tool **PGAdmin**. Carefully check that:
  - All the tables, primary and secondary keys, views, and constraints, which are defined into the *schema.sql* script file, should be available in the service database;
  - All the initial data records from *data.sql* should be available.

### 3.5. S4 Measurement Entity Class

**Figure 8:**
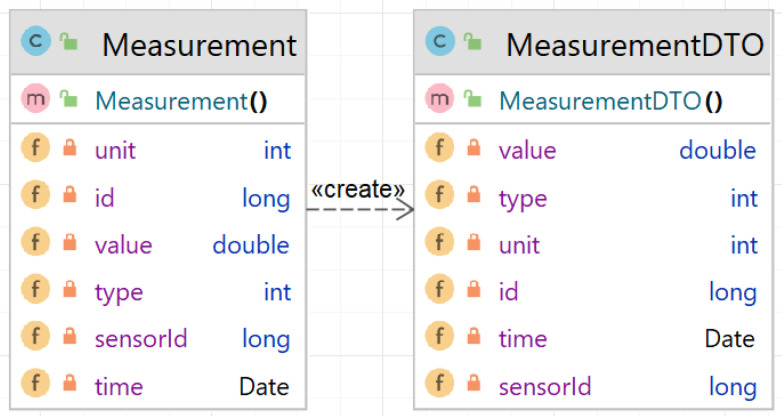
Measurement class and corresponding transport class.

The entities represent classes, which are used in order to map the database records to the respective Java objects. They are created as simple classes and then (Figure 8), using the annotation and the Spring Boot entity scanner, they become objects that are synchronized with the database table data rows.

To create and annotate the class that assures the object relational mapping, the next steps are made. Such classes allow the manipulation of data directly as objects without much concern about the database layer.

(1) Add the entities’ package: *com.unitbv.ro.MyMeasurementService.data.entity*;

(2) Create a class for each entity (for each table). As an example, the class for the entity Measurement (Listing A3);

(3) Add the annotation for the entity: *@Entity, @Table, @Column* (Listing A4);

(4) Add the Getter and Setter methods for the class member variables.

These can be added in Eclipse by accessing the menu Source− >Generate Getters and Setters.

It should be noted that these accessors are public.

### 3.6. S5 Create Repository

The repositories are small units of work proposed by the framework for working with entities in order to manage the data.

(1) Define a package for repositories: *com.unitbv.ro.MyMeasurementService.data.repository*;
(2) Create a new interface as a repository for working with measurement entity objects (Listing 2);

**Listing 2.** Interface for measurements repository.public interface MeasurementRepository {}

(3) Extend the interface to use CrudRepository as base class (Listing 3);

**Listing 3.** Use of CrudRepository to extend measurements repository.public interface MeasurementRepository extends CrudRepository <Measurement, String> {}

(4) Set the interface annotation to *@Repository*;
(5) Declare one of the first methods of the repository class, e.g., *findBySensorId* (Listing 4).

**Listing 4.** Declare findBySensorId method of the measurements repository@Repository**public interface** MeasurementRepository **extends** CrudRepository <Measurement, String>{ List <Measurement> findBySensorId (**long** id);

### 3.7. S6 Adding the Business Logic Service

These types of work units are responsible for the implementation of the business logic:

(1) Add the package: *com.unitbv.ro.myMeasurementService.bl.service*;
(2) Define a service that handles measurements related functionalities (Listing A5);

The services consider one or more repositories in order to implement functionalities, which are based on multiple repositories;

(3) Create data transport object (DTO) class for measurement (Listing A6);

(4) Extend the service with the method for retrieving the measurements corresponding to a given device from the database *getMeasurementsForDevice(long deviceId)* (Listing A7).

At the service level, the functionalities usually require more data about the measurement that is acquired from multiple database tables (or multiple entities) by using the proper repositories. The data about the measurement are gathered and sent further to the graphical user interfaces, or used for other purposes (e.g., compute how many times a measurement was borrowed). The availability of these data is provided by some data transport objects. In this case, the *MeasurementDTO* class is used.

### 3.8. S7 Define Controller That Handles the Service Functionalities Requests

(1) Add package: *com.unitbv.ro.myMeasurementService.web.application*;
(2) Add controller class *MeasurementWebController*. The controller is a class that manages the connection between the interface components and the data model;
(3) Set controller annotations (Listing 5);

**Listing 5.** Controller class with request mapping annotation.@Controller@RequestMapping (value = "/measurements")

**Figure 9:**
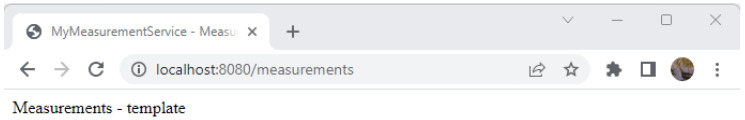
Measurements web page.

(4) Add a method that initially returns a simple string into controller (Listing A8);

(5) Add the web page measurements.html into *static∖template* and the code to display an initial message (Listing A11);

(6) Access and test the new page http://localhost:8080/measurements (Figure 9);

(7) Extend controller to publish the measurements using the *jsf-view/xhtml* (Listing A9).

### 3.9. S8 Creation of a Simple UI for Direct Service Access Using *Thymeleaf*

**Figure 10:**
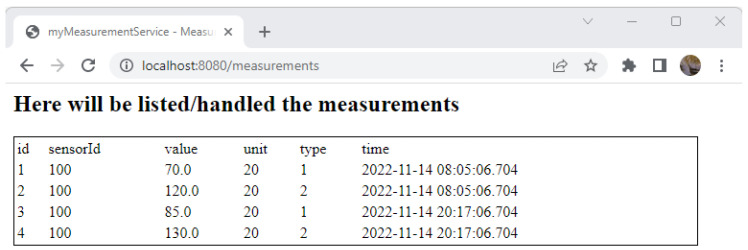
List of measurements.

(1) The *Thymeleaf*-based interface is extended to use the data received from the controller using the *Model* (Listing A12);

(2) Reload and test http://localhost:8080/measurements and see the list of measurements that exists into the database (Figure 10).

### 3.10. S9 Embedded Server Port Configurations

An immediate and useful configuration that can be made regarding the settings of the embedded server, which is used by the Spring Boot application, is to change its communication port. The modification of the relevant configuration parameters represents a powerful tool, which should be considered when integrating the required microservices:

1. The default configuration file, *application.properties*, was generated together with the project skeleton and it should be available in the folder main∖resources, otherwise, it can be manually created;
2. Add a line with server port: *server.port = 8000*;
3. Restart the application (and/or web container);
4. Re-access the application into the browser using the new port http://localhost:8000;
5. If the modification was applied for test purposes, undo the port change by removing the *server.port = 8000* configuration line and restart.

### 3.11. S10 Define RESTful Web Service Controller

The main possibility to access and consume the microservice is to access its published endpoints. The data from the database are retrieved and consumed using other web services. These operations are changing data that are provided in the form of strings or JSON representations of the respective data structures. In this case, the graphical user interface is not mandatory. The evaluation of the HTTP requests and responses may consider third-party tools, such as Postman or the rest client that is provided as an Eclipse plug-in.

(1) Add package: *com.unitbv.ro.MyMeasurementService.data.webservice*;
(2) Add controller class, *MeasurementRestController* (uses *RestController* annotation and it processes RESTful web requests, Listing A10);

(3) Restart the application (e.g., *mvn spring-boot:run*);
(4) Access the web service using the following URL http://localhost:8080/getmeasurement.

**Figure 11:**
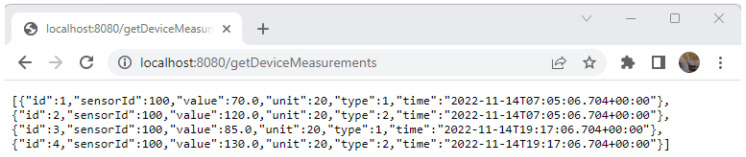
Access the web service getmeasurement.

Thus, Figure 11 displays the returned JSON format for the measurements record, which corresponds to the sensor with id 100. This was hard set as default inside the method.

findDeviceMeasurements of the MeasurementRestController using Optional;

(5) Modify the method call to use the GET parameter of the URL so that the web service is accessed with parameter value;

(6) Restart the application (e.g., *mvn spring-boot:run*);
(7) Access the web service using the following URL
  http://localhost:8080/getDeviceMeasurements?deviceId=300.

**Figure 12:**
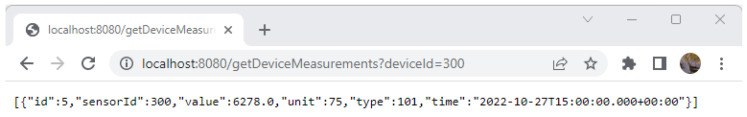
Access the web service endpoint using an HTTP GET parameter.

In Figure 12, the request parameter is read and forwarded to the JPA methods, and afterwards, the JSON result, which is represented by the monthly smart meter reading for energy consumption, is sent to the browser as a JSON response.

## 4. Authentication Microservice

The authentication mechanism, in the case of legacy applications, is based on the server, which *keeps the secrets* and, when it is queried, the provided credentials are validated, and the access authorization is provided. The server keeps track of login and session information in order to prevent relevant cybersecurity problems, such as the replay attack. Often based on the clients’ needs, the provided tokens are stored during their working sessions (e.g., as cookies). The microservice authentication process implements different mechanisms due to their distribution and asynchronous model of action. A widespread method that is used for authentication is JSON Web Token (JWT). The method enables the delegation of authentication to external services in a stateless and space-efficient manner [61]. JWT implements other standards, such as JSON Web Signature (JWS) or JSON Web Encryption (JWE), for message authentication and encryption.

A JWT consists of three parts: header, payload, and signature. Let us note the example provided in the RFC specification and listed in Table 1. The header section indicates that the media type is *application/jwt* and specifies the algorithm used for digitally signing the token, in this case, HMAC SHA-256.

The payload section is used for storing the claims. Claim Names are case-sensitive and have to be unique. Otherwise, applications can reject the JWT or use the lexical last claim name as a fallback.

For interoperability purposes, some names are registered in the IANA *JSON Web Token Claims* registry. These include the issuing entity (*iss*), the issue date and time (*iat*), as well as the expiration date (*exp*). The last claim from the payload section in Table 1 represents a custom claim, which signifies that the user successfully authenticated as an administrator on the specified domain, and services can use it for authorization purposes.

To build a complete JWT, the contents of the different sections will be encoded with base64url and concatenated with separating dots (Table 2).

According to the specification, the JWT implementations only have to include the signature algorithms HMAC SHA-256, the other fields being optional.

Considering an application that implements microservices-based distributed architectures, a microservice could handle the login process and provide a JWT that would be stored on the client side. The validity of the JWT could then be checked either on the API Gateway or by each microservice that receives a request.

When connecting to the user interface, the application detects that the user possesses no valid authentication token and redirects them to the login page. After entering the credentials, a request is sent to the */user/login* path, which contains the email and password in the form of JSON records. The ingress controller identifies all URLs starting with */user* as belonging to the authentication service and consequently routes them accordingly. The authentication service checks the credentials against the ones stored in the directory (or database), and if valid, it issues a pair of authentication and refresh tokens.

**Figure 13:**
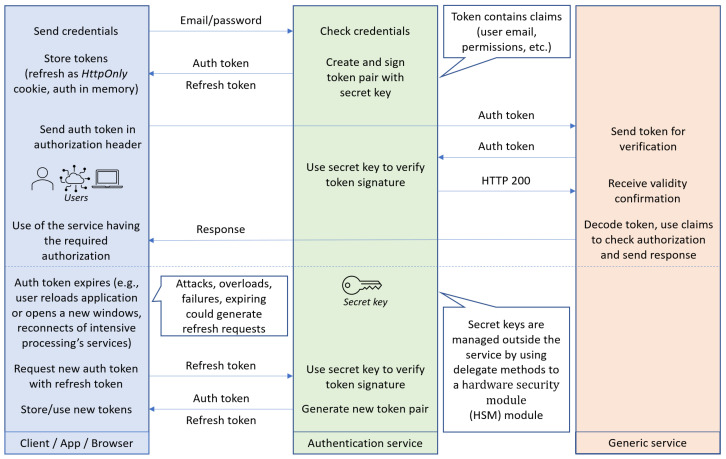
Authentication token sequence.

Using the valid tokens, the user is considered to be logged into the whole application and can use the other microservices (Figure 13). Firstly, the user is forwarded to a dashboard application, where they can access the other functionalities using the specific menu and options. A request containing the newly acquired JWT in the authorization header is sent to the accessed service at the location */serviceroute*. Before sending the response, the service must first check the validity of the authentication token. In the background, the JWT is sent to the authentication service for verification. If the token is valid, the service proceeds to reply with the requested data using a JSON format.

## 5. IoT Microservices Security Aspects

The design and development of microservices that also wrap up the use of IoT devices and their communication with complex architectures were discussed, considering different optimization aspects in the existing literature. In [62], the authors have developed an algorithm for the enhanced discovery and reuse of object requests so that the searching for objects instances is optimized by reducing the access time. When properly implemented and used, such features offer more performance and increased reliability to any IoT-enabled platform. Despite their importance, some of these optimizations are hidden in the implementation of the considered software frameworks, which are represented by context and dependency injection mechanisms, related concepts and benefits. The reusability of instances relates to relevant safety aspects and can further be connected to the cybersecurity aspects of the relevant architectures and platforms. For the specific case of an IoT system, appropriate research methods, for example, learning-based methods [63], have to be applied to treat the difficulties faced by IoT devices after the occurrence of a cyberattack.

The case study for the architecture experimented on by the authors and presented in this paper was the use of smart metering devices, which are related to energy consumption. In simple terms, the scenario implies that the clients of such a platform have to access their consumption in real-time using the proper web interfaces, and at the same time, the energy provider can do estimations concerning the consumption for its entire network of clients, while respecting all the regulations that pertain to the tariffs. Nevertheless, the energy provider can be different from the smart meter provider and also from the transport company in such a way that a heterogeneous combination of stakeholders can read or write information into the systems. It implies that the security specifications and requirements are clearly defined concerning the smart metering PKI, the encryption algorithms, security modules, protection profiles, and administration, together with the respective operations and processes [64].

**Figure 14:**
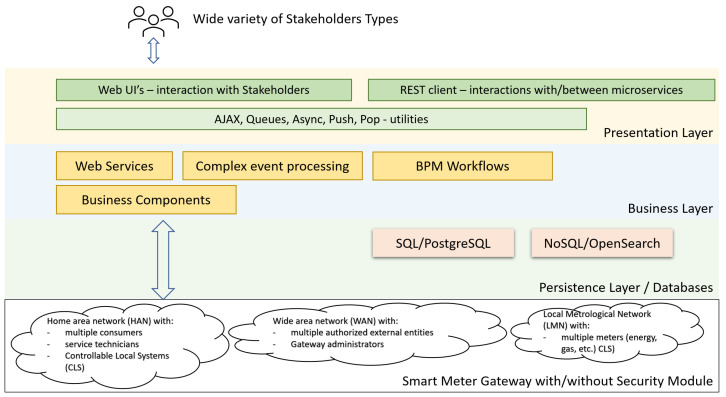
Smart meter architectural context.

Figure 14 represents the context architecture for the whole ecosystem of smart meter gateways. Thus, it considers the proper microservices-based architecture, which is represented in Figure 1. This is oriented on a single data source service. The operation of a smart meter implies a multitude of related microservices. Essentially, a smart meter gateway is placed in front of a single consumer and a single electricity meter. Even so, the stakeholders that need access to the measurement data and also to the relevant reading context are different. The data that are collected by the enrolled electricity meters are transmitted in a fully encrypted form over insecure data transmission channels.

Similar to other types of intelligent devices, the updates represent a usual process which must be secured. The updates must not affect the actual electricity meters data collection processes. First, the new system has to be uploaded on the meter, and then the updated firmware should be triggered considering the chosen time intervals.

One of the simplest optimal ways to deal with the smart meter gateway-related processes is to encapsulate its functionalities into separate microservices. As an example, considering the readings a microservice performs, the collected data are packed into a specific telegram that is valid within the platform. The telegram is published using the platform queues and consumed by other microservices as needed in order to provide their functionalities. The telegram is encrypted using the certificates installed on the smart gateway and agreed with the platform. Each time, when it is required, the exchanged telegram between platform components can be verified by the certification authority.

The effective real-world demand, not only for smart meter types such as intelligent thermostats and electricity meters, but also for other kinds of smart meters, is rapidly growing on the market. Together with this objective market increase, the theoretical and empirical research results determine the related hardware and software progress. For example, in Germany, one in ten (10%) households use smart meters, which amounts to 3.7 million households out of the overall 40.7 million [65]. Thus, private homes need to manage and control their smart metering systems through online channels in order to save energy. The increasing energy prices and the need to avoid shortages place this market on an ascending trend (Table 3).

Smart Grids spread across countries need to be reliable in terms of offering stable and scalable services and need to be secured against increasing security threats and attacks [66]. Let us recall that smart meters also measure gas and water consumption, in addition to electricity. The cumulative number of smart meters is increasing, and these IoT devices need to be managed, controlled, and queried using intelligent software architectures. The system architecture that is proposed in this paper represents one of the few integrated solutions which fulfils all the necessary logical and functional constraints.

The (auto)scaling of the microservices and concurrent consuming the information provided by the contained IoT devices is an undoubted advantage. To keep the services available, further security aspects, such as throttling (avoiding capacity being exhausted by denying some traffic to avoid denial of service attacks, manage quotas) and rate limit (avoiding bursts of traffic which can cause degradation or the outage of service), were considered.

## 6. Discussion

The microservices-based approach succeeds at pointing out the boundaries between different components. A software architect or even developer would have few or no problems identifying what the functionalities offered by a service are. Thus, the separation of concern was defined by properly designing the services’ responsibilities. Additionally, fault tolerance represents another advantage.

Architectural loose coupling of the microservice containers allows their replacement and restart without negatively impacting the other services. The poorly engineered services are easily replaceable during development, while the rest of the application continued to operate as expected. Deploying changes can be as easy as pushing a commit to the master branch if Kubernetes is set up to check for newer versions of the Docker images automatically. Another advantage comes from reusability. The authentication service could easily be used in a different application without further modifications. Implied data are also isolated at the database level and can be used or backed up independently. Containerization means that the services can be easily deployed using commercial cloud infrastructures, as well. Adding features might prove to be simpler than working with monolithic applications while requiring only the development of a new microservice instead of modifying the existing ones and taking the risk of introducing new bugs. Moreover, the web interface can be multiple-sided, covering different components of the ecosystem that manage data readings, end clients, smart meters, and so on. Under normal circumstances, each team that develops a microservice should be responsible for the entire developed microservices-based architecture. This approach is also recommended for complex applications. Microservices-based architectures are better suited for complex applications, which require a high degree of flexibility and scalability. Indeed, it is often recommended to start with a monolithic structure and consequently break it up into microservices only when and if the need arises.

Designing a system around microservices might prove to be a more difficult architectural task than designing standard applications. New and exciting technologies, such as containers and orchestration layers, help the design, development, and deployment processes.

Multiple token-based standards were developed, such as OAuth, OpenID Connect, and JSON Web Token. The token-based authentication models cover the two main phases of the service requests, namely, the initial authentication and also the refresh (update) of the token. According to the architectural considerations and principles introduced in this paper, the services are separated, and they can be easily replaced when updated libraries are published, or new versions of security token standards are made available.

Identity providers may be represented by self-developed services, which manage their own credential databases and tokens, or they can be open-source identity stores, such as Okta, Keycloak, LDAP, or a combination of these. Their use is wrapped into customized services, or their own RESTful API can be used in order to secure the respective microservices-based architectures. Web redirect mechanisms are used to forward system requests to the authentication service using secure communication channels.

There exists a large pool of client types that are connected to microservices-based architectures, such as other services, phones, IoT devices, browsers, etc. Some of the clients are owned by particular organizations, while other clients are represented by external entities. Limited access to external clients must be granted through firewalls. Each type of client must be able to access the identity service in order to retrieve the needed token or to access the application functionalities. The access rights to a group of microservices cannot always be granted through a reverse proxy microservice. The trusted network is difficult to isolate. The management and validation of tokens should be accessible in the case of each microservice, which is part of the overall software system.

The tested access scenario is based on the usage of the API gateway that considers the Identity and Access Management (IAM) platform, with the possibility to customize the gateway or extend the IAM functionalities.

A reverse proxy pattern acts like a single-entry point towards a particular resource pool. Defined by an API gateway, it allows for the required security controls to be defined. Using certain policies, such as client quota, an API gateway can trace requests and monitor relevant performance parameters. An IAM platform provides capabilities that overlap with those offered by API gateways. One of the main advantages of the IAM platforms is represented by the existing implementations of multiple security standards, such as OAuth Primer.

## 7. Conclusions

Although some of the scientific contributions that have been reviewed propose valuable algorithmic, architectural, and practical features, they miss certain functional features, which may be considered in order to describe robust security models that evolve and may be partially used in order to impose proper access policies in the particular scope of microservices-based architectures. This paper surveys relevant research articles, which highlight valuable ideas and concepts, but also relevant drawbacks, which were analysed during the design and development of the approach that is proposed in this paper. It analytically presents and discusses security aspects concerning an integrated microservices-based system, which offers the necessary functional features and security mechanisms. The system is evaluated considering a real-world use case, which relates to the management of tens of thousands of electricity meters. This dimension determines a complex use case scenario, which presumes the efficient collection of the customers’ data, its secure transmission to the microservices-based components, as well as the optimized timely processing of the collected data using the relevant microservices-based software modules. The thorough real-world performance assessment demonstrates that the proposed microservices-based architecture is capable of properly managing the enrolled electricity meters, and it also offers the required scalability, which would allow the enrolment of additional customers to occur without significant practical issues.

The authors of this paper are members of the “High Performance and Cloud Computing” research group from the Transilvania University of Brasov, Romania. This group is concerned with scientific research topics that pertain to distributed systems, cybersecurity, and IoT. Therefore, the continued development of the proposed microservices-based system is among the conceptual and practical scientific priorities of this research group.

## Figures and Tables

**Table 1:** Anatomy of a JWT.

Section	Payload
Header	{"typ":"JWT", "alg":"HS256"}
Payload	{"iss":"hpcc", "exp":1672444800, "http://cs.unitbv.ro/is_root":true}
Signature	Iuu5xStItPU5at/Nkvme4V6IfWUWZmTl0bX0EimXYxo

**Table 2:** Sample base64 encoded JWT section.

JWT Token Section	
eyJ0eXAiOiJKV1QiLA0KICJhbGciOiJIUzI1NiJ9	Header
.	
eyJpc3MiOiJocGNjIiwgImV4cCI6MTY3MjQ0NDgwMCwgImh0dHA6Ly9jcy51bm	Payload
l0YnYucm8vaXNfcm9vdCI6dHJ1ZX0	
.	
Iuu5xStItPU5at/Nkvme4V6IfWUWZmTl0bX0EimXYxo	Signature

**Table 3:** Household equipped with smart devices and systems, 1 January 2022.

Smart Device Type	% Net Income under 2500	% Net Income 2500 to under 18,000
Smart TV	46	71
Smart Speakers	9	21
Smart household appliance	8	18
Smart energy management	5	14
Smart security system	7	12

## Data Availability

Not applicable.

## Abbreviations

    The following abbreviations are used in this manuscript:

5G	Fifth Generation Mobile Network
5GsatEC	5G Satellite Edge Computing
AIMS	Artificial Intelligence as a Microservice
CPU	Central Processing Unit
C-RAN	Cloud Radio Access Network
DNN	Deep Neural Networks
DNP3	Distributed Network Protocol 3
FPGA	Field-Programmable Gate Array
GPU	Graphics Processing Unit
HTTP	Hypertext Transfer Protocol
IANA	Internet Assigned Numbers Authority
IAM	Identity and Access Management
ICS	Industrial Control Systems
IDPS	Intrusion Detection and Prevention System
IDS	Intrusion Detection System
IoT	Internet of Things
ITS	Intelligent Transportation System
JPA	Java Persistence API
JSON	JavaScript Object Notation
JWT	JSON Web Token
MSA	Microservices Architectures
NF	Network Functions
OAuth	Open Authorization
ROMA	Resource Orchestration for Microservices-based 5G Applications
SOA	Service-Oriented Architecture
SCADA	Supervisory Control and Data Acquisition
SDN	Software Defined Networks
SLO	Service Level Objectives
TCP	Transmission Control Protocol
QUIC	Quick UDP Internet Connections
UI	User Interface
UPF	User Plane Function
VNF	Virtual Network Functions
VM	Virtual Machine

## Appendix A. SQL Code Samples

**Listing A1.** *schema.sql*—Data definition language script file with database schema.	
**drop table** measurements;	
**create table** measurements (	
measurement_id	serial **primary key**,
sensor_id	bigint,
**value**	**numeric** (8, 2),
unit	**smallint**,
type	**smallint**,
measurement_time	**timestamp**
);	

**Listing A2.** *data.sql*—Script file with sample data.				
**INSERT INTO** measurements ( sensor_id, **value**, unit, measurement_type, measurement_time)				
**VALUES**				
(100,	70.00,	20,	1,	’2022−11−14⎵08:05:06.704’),
(100,	120.00,	20,	2,	’2022−11−14⎵08:05:06.704’),
(100,	85.00,	20,	1,	’2022−11−14⎵20:17:06.704’),
(100,	130.00,	20,	2,	’2022−11−14⎵20:17:06.704’),
(300,	6278.00,	75,	101,	’2022−10−27⎵17:00:00.000’);

## Appendix B. Java Code Snippets

**Listing A3.** Measurement class.
**public class** Measurement {
**private long** id;
**private long** sensorId;
**private double** value;
**private int** unit;
**private int** type;
**private** Date time;
}

**Listing A4.** Measurement class with JPA annotations.
@Entity
@Table (name = "measurements")
**public class** Measurement {
@Id
@Column (name = "measurement_id")
**private long** id;
@Column (name = "sensor_id")
**private long** sensorId;
@Column (name = "value")
**private double** value;
@Column (name = "unit")
**private int** unit;
@Column (name = "measurement_type")
**private int** type;
@Column (name = "measurement_time")
**private** Date time;
}

**Listing A5.** Spring Boot Measurement Service.
@Entity
**public class** MeasurementService {
**private final** MeasurementRepository measurementRepo;
@Autowired
**public** MeasurementService (MeasurementRepository measurementRepo) {
this.measurementRepo = measurementRepo;
}
}

**Listing A6.** Data transfer object for measurement instances.
**public class** MeasurementDTO {
**private long** id;
**private long** sensorId;
**private double** value;
**private int** unit;
**private int** type;
**private** Date time;
**public long** getId () {
**return** id;
}
**public void** setId (**long** id) {
**this**.id = id;
}
...
}

**Listing A7.** Measurement service method for getting the device measurements.
@Service
**public class** MeasurementService {
**private final** MeasurementRepository measurementRepo;
@Autowired
**public** MeasurementService (MeasurementRepository measurementRepo) {
**this**.measurementRepo = measurementRepo;
}
**public** List <MeasurementDTO> getMeasurementsForDevice (**long** deviceId) {
List <Measurement> measurements = **this**.measurementRepo.findBySensorId (deviceId);
List <MeasurementDTO> measurementDTOList = measurements
.stream ()
.map (Measurement::toDTO)
.to List ();
**return** measurementDTOList;
}
}

**Listing A8.** Controller class with mapping to method.
@Controller
@RequestMapping (value = "/measurements")
**public class** MeasurementWebController {
@RequestMapping (method = RequestMethod.GET)
**public** String getMeasurements () {
**return** "measurements ";
}
}

**Listing A9.** Web controller with sending data to UI.
@Controller
@RequestMapping (value = "/measurements")
**public class** MeasurementWebController {
**private final** MeasurementService measurementService;
@Autowired
**public** MeasurementWebController (MeasurementService measurementService) {
**this**.measurementService = measurementService;
}
@GetMapping
**public** String getMeasurementsForDevice (Model model) {
List <MeasurementDTO> measurements
= **this**.measurementService.getMeasurementsForDevice (100);
model.addAttribute ("measurements ", measurements);
**return** "measurements ";
}
}

**Listing A10.** Endpoint for microservice access.
@RestController
**public class** MeasurementRestController {
@Autowired
**private** MeasurementRepository repoMeasurements;
@RequestMapping (value = "/getDeviceMeasurements", method = RequestMethod.GET)
List <Measurement> findDeviceMeasurements (
@RequestParam (name = "deviceId", required = **false**) Long deviceId
) {
**long** sensorId = Optional.ofNullable (deviceId).orElse (100L);
List <Measurement> measurements = **this**.repoMeasurements.findBySensorId (sensorId);
System.out.println ("measurements:⎵" + measurements);
**return** measurements;
}
}

## Appendix C. Thymeleaf Sample Code

**Listing A11.** Initial static HTML5 page.
<**html**>
<**head**>
<**meta charset** = "ISO−8859−1">
<**title**>MyMeasurementService—Measurements<**/title**>
</**head**>
<**body**>
Measurements—template
<**/body**>
</**html**>

**Listing A12.** Dynamic page Thymeleaf-based for UI with connection to data model.
<**html lang** = "en" xmlns: th = "http://www.thymeleaf.org">
<**head**>
<**meta charset** = "ISO−8859−1">
<**title**>myMeasurementService—Measurements<**/title**>
</**head**>
<**body**>
<**h2**>Here will be listed/handled the measurements</**h2**>
<**table style** = "border: 1px solid black; width: 95%;">
<**tr**>
<**td**>id</**td**>
<**td**>sensorId</**td**>
<**td**>value</**td**>
<**td**>unit</**td**>
<**td**>type</**td**>
<**td**>time</**td**>
</**tr**>
<**tr th**:each = "measurement: ${measurements}">
<**td th:text** = "${measurement.id}">id</**td**>
<**td th:text** = "${measurement.sensorId}">sensorId</**td**>
<**td th:text** = "${measurement.value}">value</**td**>
<**td th:text** = "${measurement.unit}">unit</**td**>
<**td th:text** = "${measurement.type}">type</**td**>
<**td th:text** = "${measurement.time}">time</**td**>
</**tr**>
</**table**>
</**body**>
</**html**>
